# Development of a patient/proxy-reported instrument for pediatric antibiotic-associated diarrhea

**DOI:** 10.1371/journal.pone.0325436

**Published:** 2025-06-04

**Authors:** Samaneh Khanpour Ardestani, Joan L. Robinson, Hsing Jou, Levinus A. Dieleman, Hien Q. Huynh, Sunita Vohra

**Affiliations:** 1 Department of Pediatrics, Faculty of Medicine & Dentistry, University of Alberta, Edmonton, Alberta, Canada; 2 Department of Medicine, Faculty of Medicine & Dentistry, University of Alberta, Edmonton, Alberta, Canada; Universitätsklinikum Magdeburg: Universitatsklinikum Magdeburg, GERMANY

## Abstract

**Objective:**

To develop and validate a patient/proxy-reported measure of the incidence and severity of pediatric antibiotic-associated diarrhea (PAAD) in inpatient and outpatient settings.

**Methods:**

A patient advisory group, consisting of five parents and two children, was engaged as a research partner. Instrument items were developed from three sources: relevant items from two previously validated instruments; relevant constructs from a newly developed core outcome measurement set; and outcomes identified by parents and clinicians as being the most important. In a prospective observational study, children (birth to 17 years old) newly prescribed antibiotics or on antibiotics for ≤ 7 days, were included and assessed at the time of presentation and daily thereafter until two weeks after antibiotic therapy was completed. Internal consistency and convergent validity of the instrument were examined.

**Results:**

Of 78 patients who agreed to participate and met the eligibility criteria, 30(38%) were lost to follow-up; Data from the remaining 48 were analyzed. By applying four different definitions of diarrhea, we found a broad range of incidence risks of PAAD (27%−83%). PAAD was more likely to develop in younger age groups (≤ 3 years old). Cronbach’s α for the severity scale was less than 0.7. A high correlation was found between the PAAD severity score and numerical rating score of diarrhea severity reported by parents (r > 0.5).

**Conclusion:**

The PAAD instrument is the first designed to measure the incidence and severity of PAAD. The instrument has content and construct validity. For reliability analyses of the severity scale, larger studies are required.

## 1. Background

Interventional studies of pediatric acute diarrhea have used heterogeneous outcome measures, often with poor reporting of their measurement properties [1]. Use of different definitions and measures, or use of measures that lack sound measurement properties, in trials with similar primary outcomes hampers comparison of results and knowledge synthesis [1,2].

Antibiotic-associated diarrhea (AAD) is a complication of antibiotic use, likely due to resulting dysbiosis [3]. Clinical trials on prevention of AAD have mainly used probiotics as the intervention. A recent review reporting the outcomes related to pediatric antibiotic-associated adverse events in probiotic trials showed that diarrhea was only clearly defined in 21 of 37 studies. Among these 21 studies, 16 different definitions of diarrhea were documented [4]. Additionally, in a previous systematic review [5], we showed that there is a disturbing lack of evidence evaluating the validity and reliability of the most commonly used pediatric diarrhea severity scales.

The Consensus Group on Outcome Measures Made in Paediatric Enteral Nutrition Clinical Trials was established in 2012 to reach consensus on common definitions for relevant outcome measures, including acute diarrhea [6]. They developed a core outcome set (COS) and a core outcome measurement set (COMS) for clinical trials evaluating strategies for prevention and treatment of pediatric acute diarrhea and gastroenteritis [7,8]. Although their work was novel and valuable, they did not derive a specific definition or measurement instrument for AAD.

Since existing instruments were designed to measure pediatric acute diarrhea or gastroenteritis and not specifically AAD, the primary objective of this study was to develop and validate a patient/proxy-reported measure of the incidence and severity of pediatric AAD in inpatient and outpatient settings, the Pediatric Antibiotic-Associated Diarrhea (PAAD) instrument.

## 2. Methods

This study was approved by the University of Alberta Health Research Ethics Board. Written informed consent was obtained from parent/guardians of all participants, and assent was obtained from children old enough to write their name.

### 2.1 Derivation of the PAAD instrument

**Aim of the instrument:** To determine the incidence and severity of AAD in children (birth to 17 years of age) who were prescribed antibiotics in inpatient and outpatient settings.

#### 2.1.1. Diarrhea incidence.

**Fig 1:**
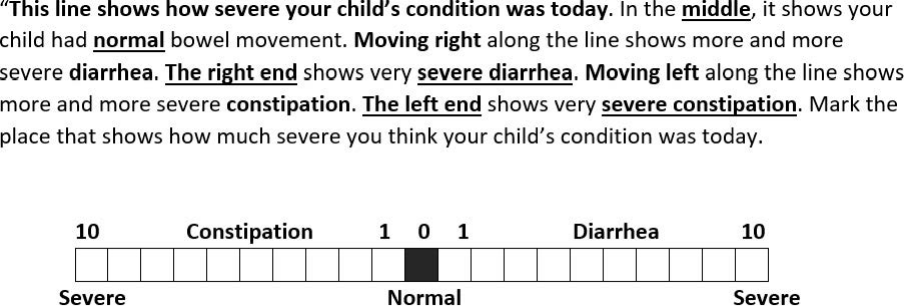
Numerical Rating Scale of bowel movement severity condition.

To determine the incidence of AAD, objective definitions of diarrhea are required. Given the previous lack of consensus, we performed a sensitivity analysis comparing the incidence of AAD in our study population using the following four common definitions to show how different definitions affect the reported incidence of diarrhea:

1. Core Outcome Measurement Set for acute diarrhea (COMS) [8]: a decrease in the consistency of loose or liquid stools and, or an increase in the frequency of evacuations, typically three in 24 hours, with or without fever or vomiting.
2. World Health Organization (WHO): the passage of unusually loose or watery stools, usually at least three times in a 24-hour period.
3. Numerical Rating Scale (NRS): a 0–10 scale where 0 indicates normal bowel movement (middle of the line) and moving to the right or left indicates progressive diarrhea or constipation, respectively (Fig 1). NRS scores of 1 on the right side indicate mild diarrhea while 10 is severe diarrhea. Daily NRS scoring reflects parental opinions without the interference of recall bias.

1. Parental global report: At the end of the follow-up period, to assess how well parental perceptions align with standardized criteria, parents/guardians were asked whether, in their opinion, their child developed diarrhea during the study period.

To assess stool consistency for the first two definitions, we used the Modified Bristol Stool Form Scale (S1 Appendix). The original Bristol stool form scale was modified first by Chumpitazi et al. (2010) [9]. The modified scale depicts 5 categories of stool consistency using drawings with descriptive captions and has high interrater (intra-class correlation coefficients (ICC)= 0.85) and intra-rater reliability (ICC = 0.87) [9] and high interobserver reliability in children 8 years and older [10]. In our study, parents of children younger than 8 years old provided proxy ratings, and we considered drawings 4 and 5 (“loose” or “watery”) in the Modified Bristol Stool Form scale to be diarrhea.

#### 2.1.2. Diarrhea severity.

To develop items for AAD severity, we reviewed the published literature on pediatric acute diarrhea measurement. To our knowledge, two more recent scales, the 20-point Modified Vesikari Score (MVS) [11] and the International Pediatric Acute Diarrheal Diseases Scale (IPADDS) [12], are the only instruments for which measurement properties have been examined. The MVS was developed in Canada [11] for outpatient settings and was validated in a US population [13]. IPADDS developed by Johnston et al (2009) and its content validity was established through a modified international Delphi study [12]. Both scales were developed to measure the severity of acute gastroenteritis (AGE) or acute pediatric diarrhea and included vomiting and fever in the final score.

To develop our PAAD instrument, we adapted relevant items from these two scales. Vomiting and fever were not included in the calculation of total score but were collected to help distinguish between AAD and AGE. We also added the relevant constructs of the recommended COS [7] and COMS [8] in order to be consistent with other studies in this area. Lastly, we included items from our previous survey [14] that parents/guardians and pediatricians identified as key PAAD outcomes: stool frequency and consistency, diarrhea duration, prevention of dehydration, hospitalization, physician or emergency department visit, and disruption of normal daily activities (eating, sleeping, playing).

#### 2.1.3. Final components of PAAD instrument.

Initially, we developed different PAAD instruments for inpatient and outpatient settings.

In outpatient settings, AAD severity was assessed by diarrhea duration and frequency, physician and/or nurse practitioner visits as a substitution for the dehydration item (adapted from MVS), the need for rehydration treatment (adapted from MVS) and ability to participate in normal daily activities (adapted from IPADDS). In inpatient settings, AAD severity was assessed by diarrhea duration and frequency, the need for rehydration treatment and estimated prolongation of hospital stay due to AAD.

Ultimately, we decided to use the outpatient form for all participants as none of the inpatients were treated for dehydration or had their hospital stay prolonged by AAD.

A committee of experts in clinimetrics, clinical epidemiology, general pediatrics, pediatric emergency medicine, pediatric infectious disease and pediatric gastroenterology approved the final structure and content of the PAAD instrument (S1 Appendix).

### 2.2. Patient engagement

In the design stage of this study, five parents and two children who were diverse in gender, education and ethnicity were engaged as part of an advisory council. They were recruited through patient/family registry invitations and word of mouth and interacted through in-person meetings and email communications with the academic research team. The items of this instrument were selected from items perceived most important to be measured in studies of pediatric diarrhea [14]. The advisory group reviewed the items, response options and formatting of our measurement instrument and the data collection forms and provided perspectives on the best recruitment and follow-up strategies. Study methods and data collection forms were approved by them before deployment.

### 2.3. Validation of the PAAD instrument

#### 2.3.1. Study design, setting and population.

A prospective observational study was conducted in the Emergency Department and ambulatory clinics of a tertiary care children’s hospital in Edmonton, Canada from August 15, 2019 to March 1, 2020.

We approached parents of inpatient or outpatients, birth to 17 years old, who were newly prescribed antibiotics for any reason or who were on antibiotics for fewer than seven days at the time of presentation. We asked for participation until 2 weeks after antibiotic therapy was finished (the time interval in which the incidence of AAD is highest).

Exclusion criteria:

1. Parental report of current diarrhea or diarrhea within the last week.
2. Anticipated antibiotic use for ≤ 2 days or at sub-therapeutic doses (e.g., prophylaxis).
3. Inflammatory bowel disease, irritable bowel syndrome or other causes of diarrhea.
4. Parent/guardians without phone or email access or who could not communicate in English.
5. No parents/guardians who could complete the baseline measurement.
6. Previously enrolled in this study.

#### 2.3.2. Administration.

The initial screening visit took place in the pediatric emergency department and ambulatory clinics of the participating hospital. After meeting the eligibility criteria and providing written informed consent, demographic information and baseline stool frequency and consistency were recorded. Parents/guardians were asked to record their child’s stool frequency and consistency daily until two weeks after antibiotic therapy was finished (S2 Appendix). For stool consistency, they were instructed to compare their child’s most abnormal stool appearance over the preceding 24 hours to the categories on the Modified Bristol Stool Form Scale. They also rated their child’s most abnormal stool using the NRS. For outpatients, an additional question regarding child’s normal activities (e.g., eating, sleeping, playing) was asked every day. Vomiting and fever were recorded by parents daily, and if either was present, the child was presumed to have infectious diarrhea rather than AAD. At the end of the follow-up period, parents/guardians were asked to report whether they thought that the child had diarrhea, the duration of diarrhea and if any physician/nurse practitioner visit or treatment were sought (S2 Appendix). For uniformity, every effort was made to have the parent/guardian form filled out by the same person. All parents/guardians were contacted daily by email or text message, based on their preference, for the duration of treatment and the next two weeks. Older children were encouraged to rate their own stool.

#### 2.3.3. Sample size.

A minimum of 50 patients is recommended to calculate correlation coefficients for construct validation. For factor analysis, a minimum of 4–10 cases per item is suggested [15]. Considering that PAAD instrument had 5 items adapted from two validated instruments, we aimed to include at least 50 patients in our study.

#### 2.3.4. Analysis.

*Content validity:* Content validity is defined as “the degree to which the content of a measurement instrument is an adequate reflection of the construct to be measured” [16]. To ensure its relevance and comprehensiveness, we developed the items in the PAAD instrument based on previous measures for which face and content validity had been established. Items were also chosen based on our previous survey of parents and clinicians [14]. Finally, a committee of experts and patient partners approved its content and structure.

*Construct validity (hypothesis testing):*

- Hypothesis testing:
  - We expected to observe high correlation (0.5 or higher) between the PAAD instrument severity score and NRS severity score (convergent validity).
  - Information about child absenteeism from day care/school and parental absenteeism from work were gathered to assess the correlation between disease severity and its impact on family life. We expected to observe high correlation (0.5 or higher) between the PAAD instrument severity score and child absenteeism from day care/school and parental absenteeism from work.

*Internal consistency*: Internal consistency is defined as the degree of correlation among items of a uni-dimensional multi-item instrument, therefore measuring the same concept [16]. We performed exploratory factor analysis to identify the dimensions of the PAAD instrument. Internal consistency was examined separately for each dimension (subscale) by Cronbach’s α [15]. Cronbach’s α between 0.70 and 0.95 is considered to indicate good internal consistency [17].

## 3. Results

### 3.1. Population characteristics

Parents of 104 children were approached from August 2019 through March 2020 of which 95 agreed to participate. Eighty-five patients met eligibility criteria, of whom seven were eventually excluded (five received antibiotics for less than 2 days, and two received polyethylene glycol (a laxative which can cause diarrhea)). No study participants were excluded for having presumed infectious diarrhea.

Of the remaining 78 participants, 30 (38%) were lost to follow-up (never responded or responded for only one or two days), hereafter referred to as “non-respondents”. The remaining 48 participants responded for at least the duration of their antibiotic therapy and were included in data analysis. The only significant difference between respondents and non-respondents was parental age (Table 1).

**Table 1:** General characteristics of 78 children enrolled in a study to validate a measure of antibiotic associated diarrhea (PAAD).

	Respondents (N = 48)	Non-respondents (N = 30)	P value
**Outpatient, n (%)**	40 (83%) outpatients	26 (87%) outpatients	0.75
**Inpatient, n (%)**	8 (17%) inpatients	4 (13%) inpatients	
Duration of hospitalization	2-7 days	1-3 days	
**Patient’s Age (years)**			
Range	1 month- 16 y	4 months-17 y	0.39
Mean (SD)	4.2 (3.9) years	5.1(5.1) years	
**Sex, n (%)**	25 (52%) Male	15 (50%) Male	0.85
**Ethnicity, n (%)**			0.09
White/ European/ Caucasian	25 (52.1%)	12 (42.9%)	
East and South East Asians	3 (6.2%)	1 (3.6%)	
South Asians	4 (8.3%)	2 (7.1%)	
Middle Eastern	6 (12.5%)	0	
Black	1 (2.1%)	3 (10.7%)	
Latin American	2 (4.2%)	0	
North American Indigenous	1 (2.1%)	3 (10.7%)	
Other (Mixed, Pacific Islander)	6 (12.5%)	7 (25%)	
**Primary diagnosis, n (%)**			0.5
Pneumonia	11 (22.9%)	9 (30%)	
Acute otitis media	6 (12.5%)	6 (20%)	
Urinary tract infection	8 (16.7%)	1 (3.3%)	
Cellulitis	9 (18.8%)	6 (20%)	
Abscess	2 (4.2%)	2 (6.7%)	
Sepsis	3 (6.2%)	0	
Animal bite	3 (6.2%)	3 (10%)	
Other (Febrile neutropenia, lymphadenitis, conjunctivitis, sinusitis, pharyngitis, balanitis)	6 (12.5%)	3 (10%)	
**Antibiotic name, n (%)**			0.07
Amoxicillin	14 (29.2%)	9 (30%)	
Amoxicillin-clavulanate	5 (10.4%)	4 (13.3%)	
Cephalosporins	21 (43.8%)	6 (20%)	
Combination of penicillin and cephalosporin class	3 (6.2%)	1 (3.3%)	
Macrolides	1 (2.1%)	5 (16.7%)	
Other (TMP/SMX, Piperacillin-tazobactam, clindamycin, other combinations)	4 (8.3%)	5 (16.7%)	
**Duration of antibiotic therapy, days**			
Range	3-28 days,	5-14 days,	0.33
Mean (SD)	8.3 (4.29)	7.5 (2.2)	
**Parent age n (%)**			**0.03**
< 20 or younger	0	0	
21-25	2 (4.3%)	3 (11.5%)	
26-30	5 (10.9%)	7 (26.9%)	
31-35	14 (30.4%)	3 (11.5%)	
36-40	16 (34.8%)	8 (30.8%)	
41-45	8 (17.4%)	2 (7.7%)	
46-50	1 (2.2%)	0	
>51	0	3 (11.5%)	
**Parent gender, n (%)**	37 (78.7%) female	20 (69%) female	0.34
**Parental education, n (%)**			0.34
Bachelor’s degree	28 (59.6%)	11 (47.8%)	
Post-secondary education without bachelor’s degree	11 (23.4%)	5 (21.7%)	
High school diploma	7 (14.9%)	4 (17.4%)	
Did not finish high school	1 (2.1%)	3 (13%)	

### 3.2. Incidence of AAD

The incidence of AAD based on the four definitions of diarrhea was as follows: The COMS definition: 40/48 (83%), the WHO definition: 24/48 (50%), the NRS definition: 37/48 (77%), and the parental global report at the end of the study: 13/39 (27%).

Gender, ethnicity, antibiotic type and duration were not risk factors for developing AAD by any of the definitions (S1 Table). We categorized children into three age groups [group 1 (0–3 years old), group 2 (4–6 years old), and group 3 (>6 years old)] as a post-hoc analysis and found the youngest age group (0–3 years old) had the highest risk of developing AAD according to three of the four definitions – parental global report was the exception (P = 0.07) (S1 Table).

### 3.3. Severity of AAD

#### Scoring.

Table 2 shows the PAAD instrument severity components. The instrument has a minimum of 0 and maximum of 15 points. To define cut-points for mild, moderate, and severe AAD, we looked at the distribution of item scores among our population. Considering that most patients scored zero in the “daily activities”, “physician/nurse practitioner visit”, and “treatment” items, we defined severity categories as follows: 0 as no-diarrhea, 2–3 as mild, 4–5 as moderate and>= 6 as severe.

**Table 2:** Pediatric antibiotic-associated diarrhea (PAAD) instrument severity components.

	0 point	1 point	2 points	3 points
Diarrhea duration, days	0	1-4	5	>=6
Diarrhea frequency (maximum number of diarrheal stools in 24 hours)	0	1-3	4-5	>=6
Daily activities	Normal	Reduced	Not able to participate	Hospitalized due to diarrhea
Physician/nurse practitioner visit	None	Outpatient	Emergency department visit	Hospitalized due to diarrhea
Treatment	None	Oral Rehydration	IV Rehydration	Hospitalized due to diarrhea

#### Distribution *of* scores.

**Fig 2:**
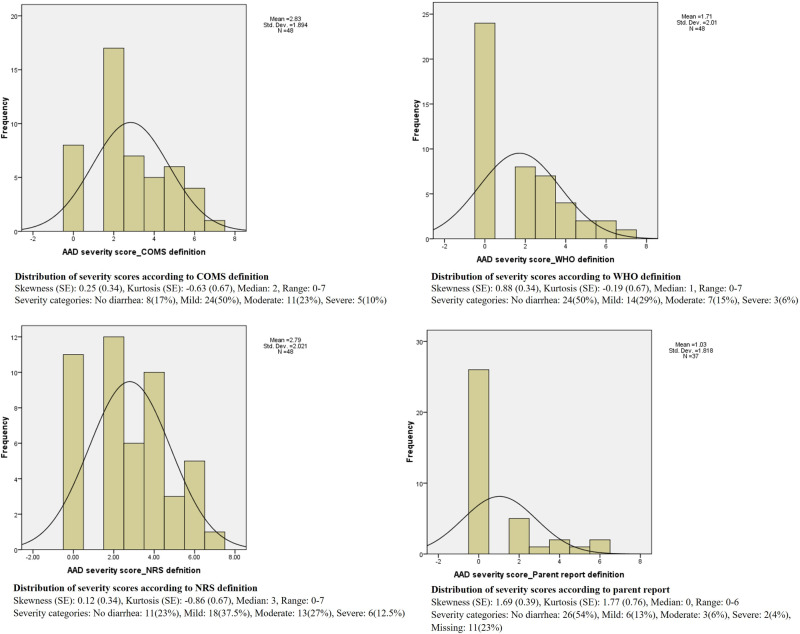
Distribution of severity scores according to different definitions.

Fig 2 shows the distribution of severity scores based on different definitions. As shown, skewness and kurtosis are minimal in COMS and NRS definitions. The severity scores distribution based on the WHO definition is slightly right skewed but still in the acceptable range. The parent reported distribution, however, is more skewed towards the right tail and kurtosis is also high, both showing less severe scores based on this definition.

#### Reliability analysis.

Most patients scored zero for “daily activities”, “physician/nurse practitioner visit” and “dehydration treatment” in the PAAD instrument. As a result, we were not able to conduct factor analysis. Cronbach’s α was less than 0.7 for all definitions, indicating low internal consistency (Table 3). In the inter-item correlation matrix (Table 4), only “diarrhea frequency” showed acceptable correlation with other items of the severity scale. The corrected item-total correlation shows the correlation between that item score and sum of the scores of the remaining items. If the correlation is more than 0.3, it shows the item can discriminate between patients with different severities and should remain in the scale [15]. Table 5 shows “diarrhea duration” and “diarrhea frequency” meet these criteria.

**Table 3:** Cronbach’s α of PAAD instrument severity score according to different definitions.

	COMS	WHO	NRS	Parent
**Cronbach’s α**	0.52	0.61	0.31	0.29

COMS: Core Outcome Measurement Set; NRS: Numerical Rating Scale, WHO: World Health Organization.

**Table 4:** Inter-item correlation matrix.

	Diarrhea frequency	Daily activities	Physician/nurse practitioner visit	Dehydration treatment	Diarrhea duration			
					COMS	WHO	NRS	Parent
**Diarrhea frequency**	1	0.25	–	0.20	0.7	0.85	0.39	0.23
**Daily activities**		1	–	−0.093	0.15	0.06	0.11	0.14
**Physician/** **nurse practitioner visit**			1	–	–	–	–	–
**Dehydration treatment**				1	0.09	0.03	0.16	0.03

Acceptable level of correlation: 0.2–0.5, more than 0.7, one could be deleted.

COMS: Core Outcome Measurement Set; NRS: Numerical Rating Scale, WHO: World Health Organization.

**Table 5:** Corrected item-total correlation for items of the severity scale according to different definitions.

	Corrected item-total correlations			
	COMS	WHO	NRS	Parent
**Diarrhea duration**	0.67	0.78	0.39	0.24
**Diarrhea frequency**	0.72	0.87	0.41	0.26
**Daily activities**	0.19	0.14	0.16	0.2
**Dehydration treatment**	0.13	0.09	0.18	0.11

Acceptable level of correlation: >=0.3.

“Physician/nurse practitioner visit” was removed as the score variance was zero.

COMS: Core Outcome Measurement Set; NRS: Numerical Rating Scale, WHO: World Health Organization.

#### Construct validity (hypothesis testing).

We observed high correlation (0.5 or higher) between the PAAD instrument severity score and worst NRS score, confirming our *a priori* hypothesis and demonstrating convergent validity (Table 6).

**Table 6:** Correlation between PAAD severity score and worst NRS reported by parents.

	COMS (n = 47)	WHO (n = 47)	NRS (n = 47)	Parent (n = 36)	Worst recorded NRS severity score (n = 47)
Worst recorded NRS severity	0.66^*^	0.6^*^	0.79^*^	0.52^*^	1

*Correlation is significant at the 0.01 level (2-tailed).

**Spearman’s rho correlation coefficients were calculated.

COMS: Core Outcome Measurement Set; NRS: Numerical Rating Scale, WHO: World Health Organization.

Since only one child missed daycare/school because of diarrhea, we were unable to analyze the correlation between the PAAD instrument severity score and child absenteeism from daycare/school and parental absenteeism from work. Out of 34 children with AAD according to the COMS definition, 19 (56%) did not miss daycare/school and the question was not applicable in the remaining 14 (41%) participants. For parents, 30/34 (88%) did not miss work and the question was not applicable in the other 4 (12%).

## 4. Discussion

To our knowledge, this is the first study to develop an instrument to measure the incidence and severity of AAD in children. Engagement of parents and children in this process was a highlight of this study. Content and construct validity, and internal consistency were examined, and promising results were obtained for the PAAD instrument.

The incidence of AAD reported by observational and clinical trials varies significantly, depending on the sample size, the definition and diagnostic criteria used. For broad spectrum antibiotics, the risk of developing AAD has been reported to be 11–40% in children [3]. Other studies report an even wider 5–62% risk [3].

In our study, despite daily recording and using a standardized validated instrument to assess stool consistency (modified Bristol stool form scale), we found a broad range in incidence of AAD using four different definitions of diarrhea. When a change in stool consistency from baseline was considered the main indication of diarrhea (COMS definition), the incidence observed was very high (83%). Conversely, only about one-quarter of parents thought that their child had diarrhea, suggesting that parents only considered it to be diarrhea if their child developed a marked change in stool consistency and/or frequency.

In contrast, applying the definition of three or more loose or liquid/watery bowel movements per day (WHO definition), showed a 50% incidence of diarrhea. Using the same definition an observational study of 75 children up to 12 years old with acute respiratory tract infections also showed a 52% prevalence of AAD [18] although a prospective study of 289 children up to 17 years old showed a 20% incidence of AAD [19].

In our study, children in the youngest age group (0–3 years old) were most likely to have AAD as shown in previous studies [18,19]. This is thought to be related to immaturity of the gastrointestinal tract and microbiota alterations in younger children [19]. Antibiotic type and duration did not affect the risk of developing AAD in our study. However, as the majority of participants were exposed to relatively few antibiotic types, our ability to comment on antibiotic type as a risk factor for AAD was constrained.

Regarding the PAAD instrument severity scores, the distributions seemed symmetrical for all diarrhea definitions except the parental global report. Consistent with results of a previous study [20], our results showed that most patients developed AAD of mild to moderate severity.

We were unable to run the reliability analyses (factor analysis and internal consistency) for the severity scale of the PAAD instrument because most of our patients scored zero for the three items of “daily activities”, “physician/nurse practitioner visit” and “dehydration treatment”.

The high correlation found between the PAAD instrument severity score and the worst NRS score confirmed the convergent validity of the PAAD instrument. However, there was insufficient data to determine the impact of AAD on family life.

Small sample size was the main limitation of our study. Although we reached the minimum number of participant required [15] despite the unexpectedly high attrition rate and restrictions in doing clinical research at the hospital due to the COVID pandemic, many references recommend 100 participants in validation studies to reflect the full spectrum of illness severity. Future larger studies with children who are more severely affected are required to enable accurate examination of the internal consistency as well as the impact of disease severity on family life.

### Clinical implications

The primary goal of the PAAD instrument is to be used in research studies. However, as this tool can quantify parental reports of diarrhea, it could also be used in clinical practice.

## 5. Conclusion

The first instrument to measure the incidence and severity of pediatric AAD was successfully designed and assessed for its measurement properties with the engagement of parents/children in the process of development. The PAAD instrument has content and construct validity. For reliability analyses, larger studies are needed, including children with more severe AAD. The current study focuses on mild and moderate cases, which represents the majority of pediatric AAD. However, we recommend future research include a wider range of severities and to explore additional dimensions of severe disease burden, such as dehydration status.

## Supporting information

S1 Appendix Pediatric Antibiotic Associated Diarrhea Measurement Instrument-Outpatient.
(DOCX)

S2 Appendix “Daily collection form” and “End of the study form”.
(DOCX)

S3 Appendix De-identified data set. (XLSX)

S1 Table Associations of participant characteristics with incidence of diarrhea according to different definitions.
(DOCX)
