# Supplementary material for: Development of a patient/proxy-reported instrument for pediatric antibiotic-associated diarrhea
Source: PLoS One. 2025 Jun 4;20(6):e0325436. doi: 10.1371/journal.pone.0325436 (PMC12136338; doi:10.1371/journal.pone.0325436)
Supplement: S1 Appendix — Pediatric Antibiotic Associated Diarrhea Measurement Instrument-Outpatient. (DOCX) [file pone.0325436.s001.docx]

**S1 Appendix. Pediatric Antibiotic Associated Diarrhea Measurement Instrument-Outpatient**

1. Stool consistency:

**“Modified Bristol Stool Form Scale”**

* Diarrheal stools (diagrams number 4 &5)

1. Maximum number of stools per 24-h period: _____ times
2. Diarrhea duration: _____days
3. Child’s daily activities (e.g. eating, sleeping, playing):
   1. Normal b. Reduced, but still present c. Unable to participate

d. Hospitalized due to diarrhea

1. Physician/nurse practitioner visits due to diarrhea:
   1. None b. Outpatient c. Emergency department visit d. Hospitalized due to diarrhea
2. Treatment:
   1. None b. Oral rehydration c. IV rehydration d. Hospitalization due to diarrhea
