# Supplementary material for: Development of a patient/proxy-reported instrument for pediatric antibiotic-associated diarrhea
Source: PLoS One. 2025 Jun 4;20(6):e0325436. doi: 10.1371/journal.pone.0325436 (PMC12136338; doi:10.1371/journal.pone.0325436)
Supplement: S2 Appendix — “Daily collection form” and “End of the study form”. (DOCX) [file pone.0325436.s002.docx]

**S2 Appendix -Daily collection form**

| **Participant code:** _____ | **Initials:** ___/___/___  F M L | **Date:** ___/___/____  dd mm yyyy |
| --- | --- | --- |
| **Most abnormal stool appearance in the last 24 hours:**  “Modified *Bristol S*tool Form Scale”  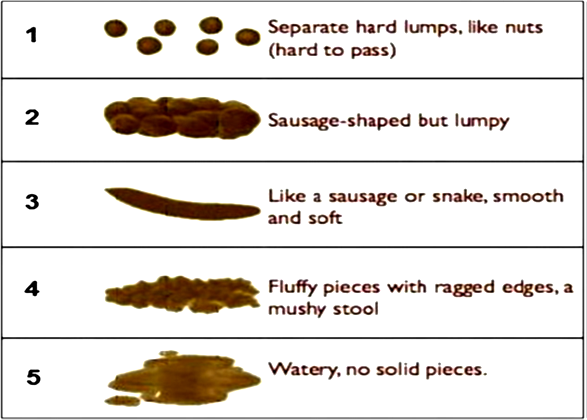  ☐ No bowel movement | | |
| **Number of bowel movements in the last 24 hours**: _____ times | | |
| **Did your child have vomiting in the last 24 hours?**  ☐Yes  ☐No  If “**Yes**”, How many times: ________ | | |
| **Did your child have fever in the last 24 hours?** | | |
| ☐Yes  ☐No  If “**Yes**”, What was the temperature: ________ | | |
| **Child’s daily activities in the last 24 hours (e.g. eating, sleeping, playing)**:  ☐ Normal  ☐ Reduced, but still present  ☐ Not able to participate at all  ☐ Hospitalized due to diarrhea | | |
|  | | |
| “**This line shows how severe your child’s condition was today**. In the **middle**, it shows your child had **normal** bowel movement. **Moving right** along the line shows more and more severe **diarrhea**. **The right end** shows very **severe diarrhea**. **Moving left** along the line shows more and more severe **constipation**. **The left end** shows very **severe constipation**. Mark the place that shows how much severe you think your child’s condition was today.  **10** **Constipation 1 0 1 Diarrhea 10**   \|  \|  \|  \|  \|  \|  \|  \|  \|  \|  \|  \|  \|  \|  \|  \|  \|  \|  \|  \|  \|  \| \| --- \| --- \| --- \| --- \| --- \| --- \| --- \| --- \| --- \| --- \| --- \| --- \| --- \| --- \| --- \| --- \| --- \| --- \| --- \| --- \| --- \|   **Severe Normal Severe** | | |

**S2 Appendix-End of the study form**

| **Participant code:** _________ | **Initials:** ____/____/_____  F M L | **Date:** ____/____/_____  dd mm yyyy |
| --- | --- | --- |
| **Diarrhea duration:** _____days | | |
| **Physician/nurse practitioner visits due to diarrhea:**  ☐ None  ☐ Outpatient (please indicate date: ________)  ☐ Emergency department visit (please indicate date: ________)  ☐ Hospitalized due to diarrhea (please indicate date: ________) | | |
| **Treatment:** | | |
| ☐ None  ☐ Rehydration (oral, nasogastric tube, intravenous- please circle)  ☐ Hospitalized due to diarrhea  ☐ Other (please specify:_______________) | | |
| **Child’s absence from school/day care due to diarrhea:** | | |
| ☐ Yes  ☐ No  ☐ Child does not attend school/day care  If “**Yes**”, How many days: _________ | | |
| **Parents’ absence from work due to child’s diarrhea:** | | |
| ☐ Yes  ☐ No  ☐ Parent does not work outside the home  If “**Yes**”, How many days: _________ | | |
