# Supplementary material for: Development of a patient/proxy-reported instrument for pediatric antibiotic-associated diarrhea
Source: PLoS One. 2025 Jun 4;20(6):e0325436. doi: 10.1371/journal.pone.0325436 (PMC12136338; doi:10.1371/journal.pone.0325436)
Supplement: S1 Table — Associations of participant characteristics with incidence of diarrhea according to different definitions. (DOCX) [file pone.0325436.s004.docx]

**S1 Table. Associations of participant characteristics with incidence of diarrhea according to four different definitions**

|  | **COMS** | | **P value** | **NRS** | | **P value** | **WHO** | | **P value** | **Parent** | | **P value** |
| --- | --- | --- | --- | --- | --- | --- | --- | --- | --- | --- | --- | --- |
|  | **AAD**  **N=40** | **No AAD**  **N=8** |  | **AAD**  **N=37** | **No AAD**  **N=11** |  | **AAD**  **N=24** | **No AAD**  **N=24** |  | **AAD**  **N=13** | **No AAD**  **N=26** |  |
| **Age of child**  **0-3 yr**  **4-6 yr**  **>6** | 24(96%)  8(61.5%)  8(80%) | 1(4%)  5(38.5%)  2(20%) | **0.010** | 24(96%)  5(38.5%)  8(80%) | 1(4%)  8(61.5%)  2(20%) | **< 0.001** | 17(68%)  2(15.4%)  5(50%) | 8(32%)  11(84.6%)  5(50%) | **0.009** | 4(22.2%)  3(25%)  6(66.7%) | 14(77.8%)  9(75%)  3(33.3%) | 0.07 |
| **Gender of child**  **Female**  **Male** | 20(87%)  20(80%) | 3(13%)  5(20%) | 0.7 | 19(82.6%)  18(72%) | 4(17.4%)  7(28%) | 0.5 | 11(47.8%)  13 (52%) | 12(52.2%)  12 (48%) | 0.7 | 4(21.1%)  9(45%) | 15(78.9%)  11(55%) | 0.1 |
| **Ethnicity of child**  **White**  **Other** | 21(84%)  19(82.6%) | 4(16%)  4(17.4%) | 1 | 21(84%)  16(69.6%) | 4(16%)  7(30.4%) | 0.3 | 15(60%)  9 (39.1%) | 10(40%)  14(60.9%) | 0.1 | 7(35%)  6 (31.6%) | 13(65%)  13(68.4%) | 0.8 |
| **Outpatient**  **Inpatient** | 32(80%)  8(100%) | 8(20%)  0 | 0.3 | 29(72.5%)  8(100%) | 11(27.5%)  0 | 0.2 | 17(42.5%)  7(87.5%) | 23(57.5%)  1(12.5%) | **0.04** | 10(31.2%)  3(42.9%) | 22(68.8%)  4(57.1%) | 0.6 |
| **Antibiotic type**  **Amoxicillin only or in combination**  **Cephalosporin only or in combination**  **Other** | 19(86.4%)  18(81.8%)  3(75%) | 3(13.6%)  4(18.2%)  1(25%) | 0.8 | 18(81.8%)  17(77.3%)  2(50%) | 4(18.2%)  5(22.7%)  2(50%) | 0.4 | 10(45.5%)  13(40.6%)  1(25%) | 12(54.5%)  19(59.4%)  3(75%) | 0.3 | 7(35%)  4(25%)  2(66.7%) | 13(65%)  12(75%)  1(33.3%) | 0.4 |
| **Antibiotic duration (days)**  **Mean±SD** | 8.8±4.5 | 6.1±2.1 | 0.1 | 8.9±4.7 | 6.7±2 | 0.1 | 9.1±5.4 | 7.5±2.5 | 0.1 | 9.7±7 | 8.2±2.5 | 0.4 |

AAD: Antibiotic-associated diarrhea, COMS: Core Outcome Measurement Set; NRS: Numerical Rating Scale, WHO: World Health Organization

*Comparisons made using independent sample T test or Chi-square.
